# Editorial: Adaptations enabling far-red light photosynthesis in oxygenic photo-autotrophs

**DOI:** 10.3389/fpls.2025.1679739

**Published:** 2025-10-31

**Authors:** Keisuke Kawakami, Yasuhiro Kashino

**Affiliations:** ^1^ Biostructural Mechanism Laboratory, RIKEN SPring-8 Center, Sayo, Japan; ^2^ Graduate School of Science, University of Hyogo, Koto, Japan

**Keywords:** far-red light, chlorophyll d, chlorophyll f, oxygenic photosynthesis, photoacclimation

Until the discovery of the oxygenic photoautotrophic cyanobacterium *Acaryochloris marina *(Miyashita et al., 1996), it was believed that the use of infrared light by photosynthesis was exclusively limited to non-oxygenic photoautotrophs (so-called photosynthetic bacteria) and oxygenic photosynthesis was driven by visible light. The major photosynthetic pigment of *A. marina* is chlorophyll (Chl) *d* whose Qy-band *in vivo* is positioned in the far-red light region. It was demonstrated through extensive investigation that *A. marina* drives oxygenic photosynthesis by utilizing far-red light whose energy level is lower than that of visible light. The use of far-red light is thought to be advantageous among a large number of Chl *a*-carrying photoautotrophs within the symbiosis host ascidians colony. It was once thought that this organism was the missing link in the evolution of photoautotrophs, situated between the far-red light utilizing non-oxygenic photoautotrophs and the visible light utilizing oxygenic photoautotrophs. However, thinking about the light environment of Archean Earth characterized by low visible light and far-red light-enriched conditions and the appearance of oxygenic photoautotrophs, it is thought that the cyanobacteria of Far-red Light Photoacclimation (FaRLiP) are suitable model organisms for the research of the origin of oxygenic photosynthesis as is pointed out by (Battistuzi et al.).

Later on, several Chl *f*-carrying cyanobacteria, such as *Halomicronema hongdechloris* isolated from stromatolite in Shark Bay located in Western Australia (Chen et al., 2010), were also discovered. These organisms can utilize longer wavelength far-red light than *A. marina*. In the interior portion of the stromatolite, in contrast to the surface, visible light is limited and only far-red light is available. Using such far-red light, *H. hongdechloris* performs photosynthesis. Other than *H. hongdechloris*, Chl *f*-carrying cyanobacteria are found in various environments. Extensive investigations have been conducted into how such far-red light drives oxygenic photochemistry with energy lower than visible light. Different from the mechanisms of Chl *d*-carrying *A. marina* in which photochemistry is driven by the energy of far-red light by the reaction center composed of Chl *d*, the photochemistry is driven in the same way as other Chl *a*-carrying photoautotrophs and Chl *f* functions as antennas. The energy acquired by Chl *f* is transferred to the reaction center by the process of uphill energy transfer. The article by Gisriel et al. in this Research Topic describes the molecular evolution of the photosystem complexes encoded in the FaRLiP gene cluster through phylogenetic analysis of PSI subunits encoded in FaRLiP.

In addition to the cyanobacterial world, it was found that the use of far-red light expands to eucaryotic photoautotrophs. *Prasiola crispa*, an aerial green alga that inhabits Antarctica, forms large layered colonies, and the cells underneath the layer utilize far-red light for photosynthesis since visible light does not penetrate the layer. Far-red light is absorbed by *P. crispa*’s unique light harvesting Chl-binding protein (Pc-frLHC) that forms ring-structure with eleven subunits and that facilitates far-red light absorption and uphill excitation energy transfer to PSII (Kosugi et al.). The 11 Chls associated with Pc-frLHC monomer are mostly Chl *a*. The absorption of far-red light becomes possible due to the red-shift of the Qy-band induced by the multimeric structure of Chls. The mechanism to absorb far-red light is different from those of Chl *d*- or Chl *f*-carrying cyanobacteria. The structure of Pc-frLHC is also unique in that it consists of four transmembrane helices while most LHC consist of three transmembrane helices. Genome analysis and phylogenetic analysis performed in the article by Kosugi et al. indicated that Pc-frLHC shares homology with Lhca genes in some *Coccomyxa* and *Trebouxia* species, which suggested Pc-frLHC evolution from one type of ancestral LHCI with four transmembrane helices.

Just a quarter of a century ago, our understanding of oxygenic photosynthesis was limited to the visible light region. However, as is summarized in this Research Topic, our understanding is now expanding to the far-red light region whose energy level is lower than that of visible light. It is of interest that the mechanisms to utilize far-red light for oxygenic photosynthesis are understood more and more, including the effect on the photosynthesis of higher plants as demonstrated by (Saeed et al.).
